# A prospective multicenter study of the efficacy of a fiber-supplemented dietary intervention in dogs with chronic large bowel diarrhea

**DOI:** 10.1186/s12917-022-03302-8

**Published:** 2022-06-24

**Authors:** Dale A. Fritsch, Susan M. Wernimont, Matthew I. Jackson, Jennifer M. MacLeay, Kathy L. Gross

**Affiliations:** 1 Hill’s Pet Nutrition, Inc., Topeka, KS USA; 2 Global Clinical Nutrition and Claims, Hill’s Pet Nutrition, Inc., P.O. Box 1658, Topeka, KS 66601-1658 USA; 3 AKC Canine Health Foundation, Inc., Raleigh, NC USA

**Keywords:** Chronic large bowel diarrhea, Dietary intervention, Dogs, Fiber, Prospective study

## Abstract

**Background:**

Chronic large bowel diarrhea is common in dogs and can have a significant impact on their overall health and well being. We evaluated the safety and efficacy of a therapeutic food with select dietary plant fibers known to contain antioxidant and polyphenol compounds on clinical signs in dogs with chronic diarrhea.

**Methods:**

A prospective clinical study was conducted in 31 adult dogs currently experiencing chronic diarrhea from private veterinary practices in the United States. Enrolled dogs were switched to a complete and balanced dry therapeutic food containing whole grains and polyphenol-containing fiber sources for 56 days. Veterinarians evaluated changes from baseline in overall clinical signs, recurrence of clinical signs, and stool parameters at Days 2, 3, 4, 28, and 56. Dog owners evaluated stool consistency daily and nausea/vomiting, quality of life (QoL), and stooling behaviors at Days 1, 14, 28, and 56. Statistical analysis was performed using a mixed-effects model with Day as a fixed-effect.

**Results:**

Assessments of overall clinical response and stool parameters indicated that diarrhea improved significantly within 1 day of initiating the therapeutic food. Veterinarians reported that 68% of dogs had complete resolution of their clinical signs by Day 56 and the remaining 32% experienced improvement (*P* < 0.05), with no cases of recurrence. Veterinarians also reported improvement in stool consistency (*P* < 0.001) and reductions of blood and mucus in stool (*P* < 0.001). Significant improvements in nausea/vomiting, stooling behaviors, and quality of life (QoL) were reported by dog owners after 28 days and were sustained through day 56 (*P* < 0.05). The therapeutic food was safe and well tolerated.

**Conclusions:**

In dogs with chronic large bowel diarrhea, the therapeutic food rapidly improved stool consistency, resolved clinical signs, and improved stooling behaviors and QoL. Therapeutic foods supplemented with fiber sources rich in antioxidant and anti-inflammatory compounds contribute to rapid resolution of chronic diarrhea without recurrence and may contribute to long term health.

**Supplementary Information:**

The online version contains supplementary material available at 10.1186/s12917-022-03302-8.

## Background

Chronic diarrhea is common in dogs and is frequently seen in patients with chronic enteropathy [1]. Chronic inflammatory enteropathy, including colitis, is considered the most common cause of chronic gastrointestinal disease in dogs, affecting up to 90% of those with chronic diarrhea of at least 3 weeks in duration [1–3].

It is widely accepted that colitis and other inflammatory bowel diseases result from a complex interplay among the intestinal microenvironment (primarily bacteria and dietary constituents), the immune system, environmental triggers, and host genetics [4–6]. In dogs with chronic colitis, secretion of mucous is excessive and absorption of water and electrolytes is impaired [7]. Contents entering the colon stimulate strong migrating muscular contractions resulting in discomfort and the urge to defecate. The most common clinical sign of chronic colitis in dogs is large-bowel diarrhea, which is characterized by tenesmus, dyschezia, increased mucus secretion, and blood, and defecations may be accompanied by pain related behaviors [7, 8]. Defecation is often urgent and frequent, with decreased fecal volume per bowel movement [7].

Most dogs with gastrointestinal (GI) disease exhibit concurrent intestinal dysbiosis, defined as an imbalance or disruption in the content or function of the intestinal microflora [9, 10]. A change in diet can result in the transit of nutrients to the colon that selectively nourish desirable colonic bacteria and provide the necessary substrate to restore healthy intestinal microbial growth, thereby reducing the symptoms of colitis [11]. In fact, results from several studies suggest that approximately 50% to 60% of dogs with chronic diarrhea due to inflammatory causes respond to nutritional interventions [1, 6, 12, 13]. Among dogs with lymphocytic-plasmacytic enteritis, 60% to 88% exhibited a positive response to dietary modification. In these reports, diets were modified to hydrolyze protein antigens or to eliminate them through use of novel protein sources. Although dietary fiber and polyphenols modify the gut microbiota community and host gut physiology, documentation of the effect of a dietary intervention with polyphenol-rich fibers is not available. While dietary interventions with modifications to the protein source or content are typically first-line therapy for dogs with chronic diarrhea [6], targeted use of dietary fibers containing plant secondary metabolites may offer ancillary benefits.

Microflora in the gut harvest energy from undigested food and concurrently produce postbiotics, which are metabolites secreted by bacteria during their digestion of bypass nutrients including fiber, plant phenolics, and protein. These include short-chain fatty acids (SCFAs), phenolic compounds originating from plants, transformed phenolytic compounds, and proteolytic products, respectively [14]. Providing a pet food with dietary fiber sources rich in antioxidants and polyphenols, compounds that are poorly digested and metabolized in the upper GI tract, supports delivery of antioxidant and anti-inflammatory compounds to the lower GI tract where they can be metabolized by the microflora in the gut to produce postbiotics [15]. Incorporating prebiotic fibers may result in expansion of the pet microbiome, leading to increased bacterial protein synthesis and increased fecal bulk. Further, expansion of microbiome activity can increase production of postbiotics, which can provide energy and exert anti-inflammatory and antioxidant effects [16–19]. Given the potential benefits of a diet with targeted prebiotic properties, a novel fiber blend was developed that consists of both soluble and insoluble fibers specifically chosen for their prebiotic content, water-holding and stool-bulking capacity, and fiber-bound plant components.

The objective of the present study was to determine if a fiber-supplemented dietary intervention known to contain antioxidant and polyphenol compounds could improve clinical signs and parameters of GI health in dogs with chronic large bowel diarrhea. A companion analysis evaluated the impact of the fiber-supplemented dietary intervention on metabolomics and the fecal microbiome (see the accompanying article by Fritsch et al.) [20].

## Results

### Demographics and study disposition

Thirty-nine dogs diagnosed with chronic large bowel diarrhea were recruited from 12 veterinary clinics across the US and a total of 31 dogs were enrolled in the study (Table 1). Some dogs also showed evidence of small bowel involvement, including vomiting and weight loss, which is indicative of enterocolitis. The mean age of the dogs included in the trial was 5.3 years (range, 1–10 years). Fifteen were female (14 spayed) and 16 were male (14 neutered). Eleven dogs were identified as mixed breeds and 20 dogs represented 13 different breeds. Five dogs failed to successfully transition onto the study food during the first week of the study and were dismissed. These dogs either ate less than 60% of maintenance food intake for 5 days, did not consume at least 60% of maintenance over 72 h, did not eat at all for more than 36 h, or did not defecate at least once a day during their stay in the clinic. Four other dogs discontinued the trial: one due to low food intake (Day 23) and three for reasons unrelated to study food, including the presence of a rectal polyp (Day 12), the presence of a previously undiagnosed heart condition (Day 6), and owner noncompliance (Day 6). A total of 22 dogs completed the 8-week study according to protocol.

**Table 1:** Demographics of study participants

Characteristic	Enrolled (*N* = 31)	Completed (*N* = 22)
**Mean Age (yrs)**	5.4	5.5
**Mean Weight (lbs)**	48.0	45.7
**Sex (No., %)**		
Male	16 (52)	12 (55)
Female	15 (48)	10 (45)
**Spayed/neutered (No., %)**	28 (90)	19 (86)
**Breed (No.)**		
Alaskan Malamute	1	1
American Staffordshire terrier	1	1
Beagle	2	2
Boston terrier	2	2
Brittany	1	1
Cocker spaniel	1	1
German shepherd	1	1
Golden retriever	1	1
Labrador retriever	2	2
Miniature schnauzer	1	1
Parson Russell terrier	1	1
Yorkshire terrier	2	2
Mixed breed	11	4
Other^a^	4	2

^a^ Akita, Chihuahua, Doberman pinscher, poodle

### Veterinary and owner assessments

**Fig. 1:**
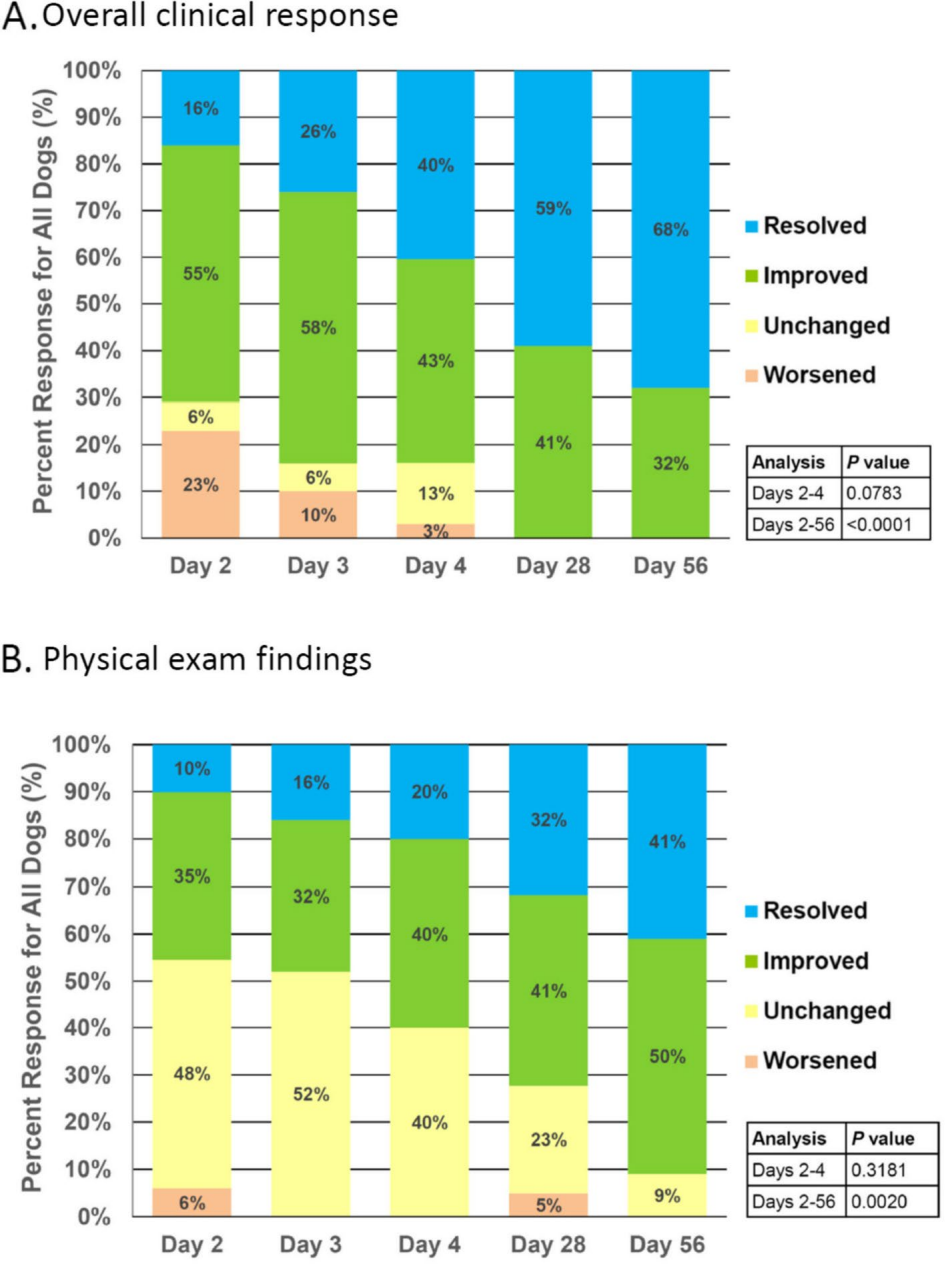
Veterinarian’s assessment of (**A**) overall clinical response and (**B**) physical exam findings. *P* values represent comparisons of the distribution of responses at Day 4 or Day 56 versus Day 2

Veterinarians reported improvements in overall clinical signs versus baseline beginning on Day 2 and continuing through Day 56 (Fig. 1A). By Day 28, veterinarians reported complete resolution in 59% of dogs and a positive response in 41% and by Day 56, complete resolution was reported in 68% of dogs and a positive response in 32% (*P* < 0.0001 vs Day 2). No relapses were reported in the 8-week study. Veterinarians also reported significant improvements in their assessments of dogs’ responses to dietary intervention through changes observed in physical exams and their overall clinical signs by Day 56 (Fig. 1B).

**Fig. 2:**
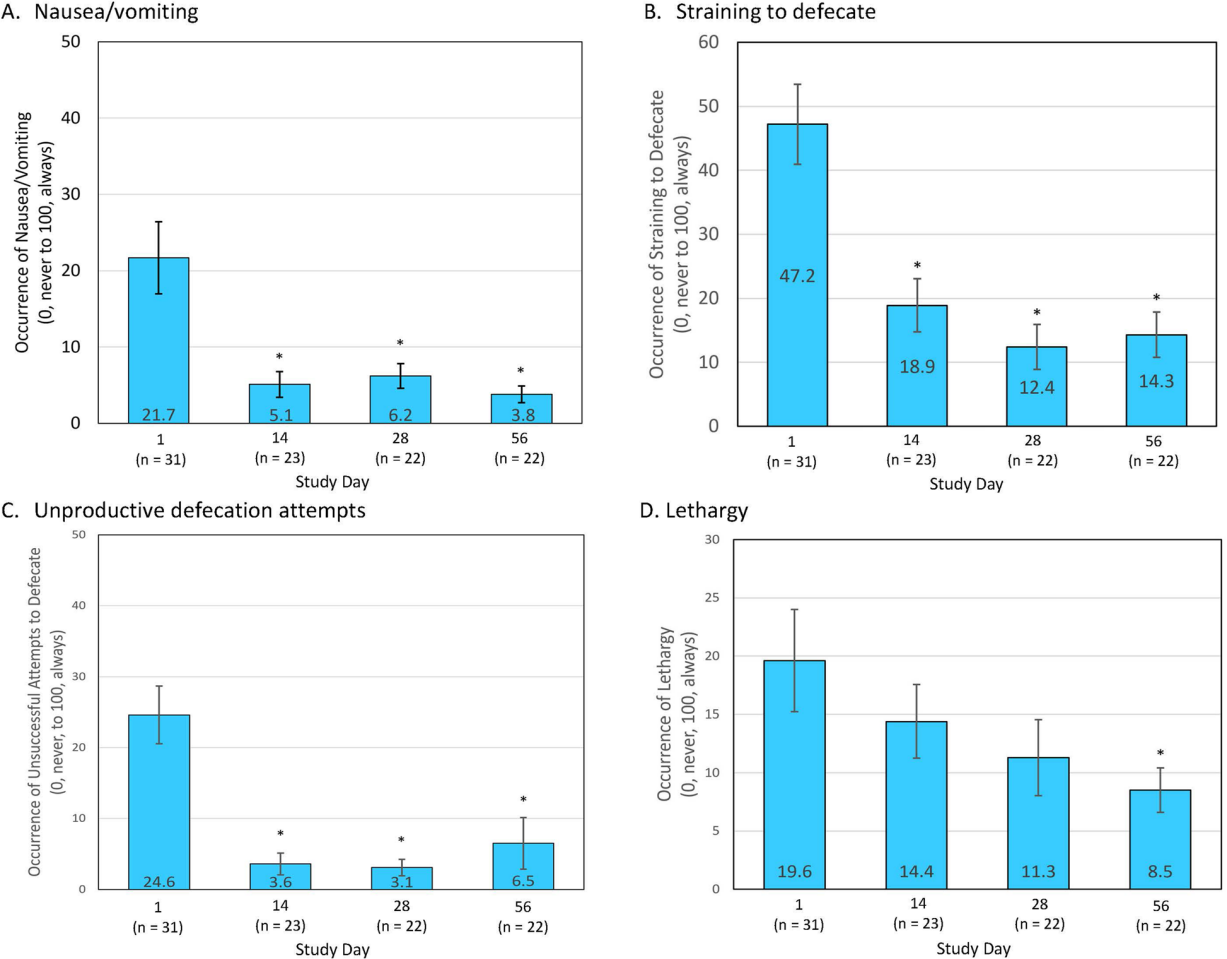
Mean frequency (± standard error) of the occurrence of clinical signs of large bowel diarrhea by dog owners’ assessment, including (**A**) nausea/vomiting; (**B**) straining while defecation; (**C**) unproductive defecation attempts; and (**D**) lethargy

Based on a visual analog scale of 0 (never) to 100 (always), dog owners observed a significant decrease in the occurrence of nausea/vomiting and adverse stooling behaviors by Day 14 and lethargy at Day 56 (Fig. 2). In addition, based on a 10-point visual analog scale, dog owners also reported a significant improvement in quality of life (QoL) from baseline (QoL score, 8.52 ± 0.32) to Day 28 (9.39 ± 0.17; *P* = 0.003) and Day 56 (9.28 ± 0.20; *P* = 0.02).

### Resolution of diarrhea

**Fig. 3:**
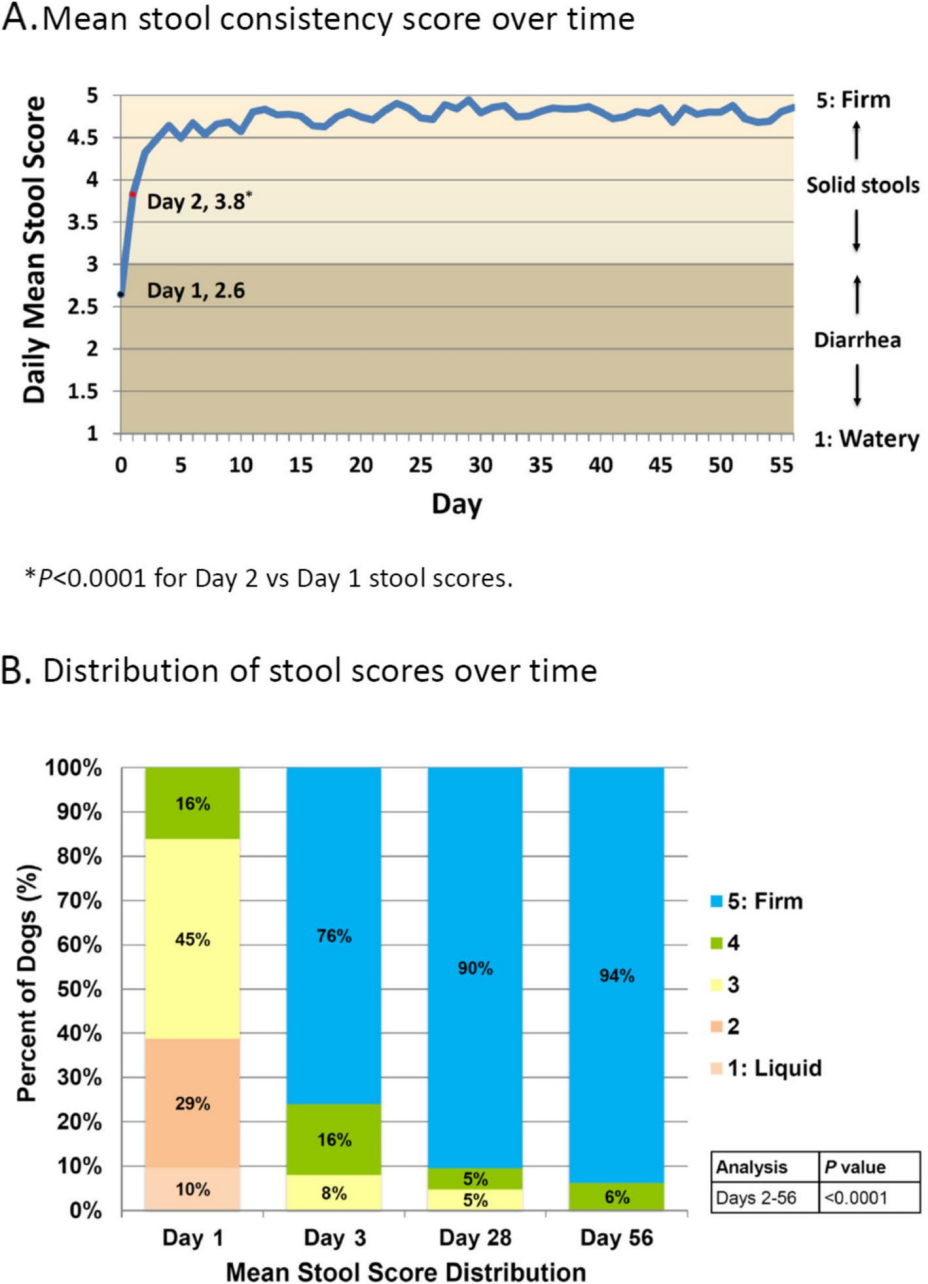
Mean stool consistency score throughout the study. Graphs show mean stool scores over time (**A**) and stool score distribution over time (**B**) as reported by veterinarians on Days 0–3 and dog owners on Days 4–56. The *P* value in (**B**) represents a comparison of the distribution of responses at Day 56 versus Day 2

Based on a 5-point scale, mean stool consistency (firmness) significantly increased from a grade of 2.6 ± 0.19 on Day 1 to 3.8 ± 0.23 on Day 2 (*P* < 0.0001). Stool consistency continued to improve through Day 4 (mean grade of 4.6) and remained at a mean grade of 4.5 or higher throughout the remainder of the study (Fig. 3).

**Fig. 4:**
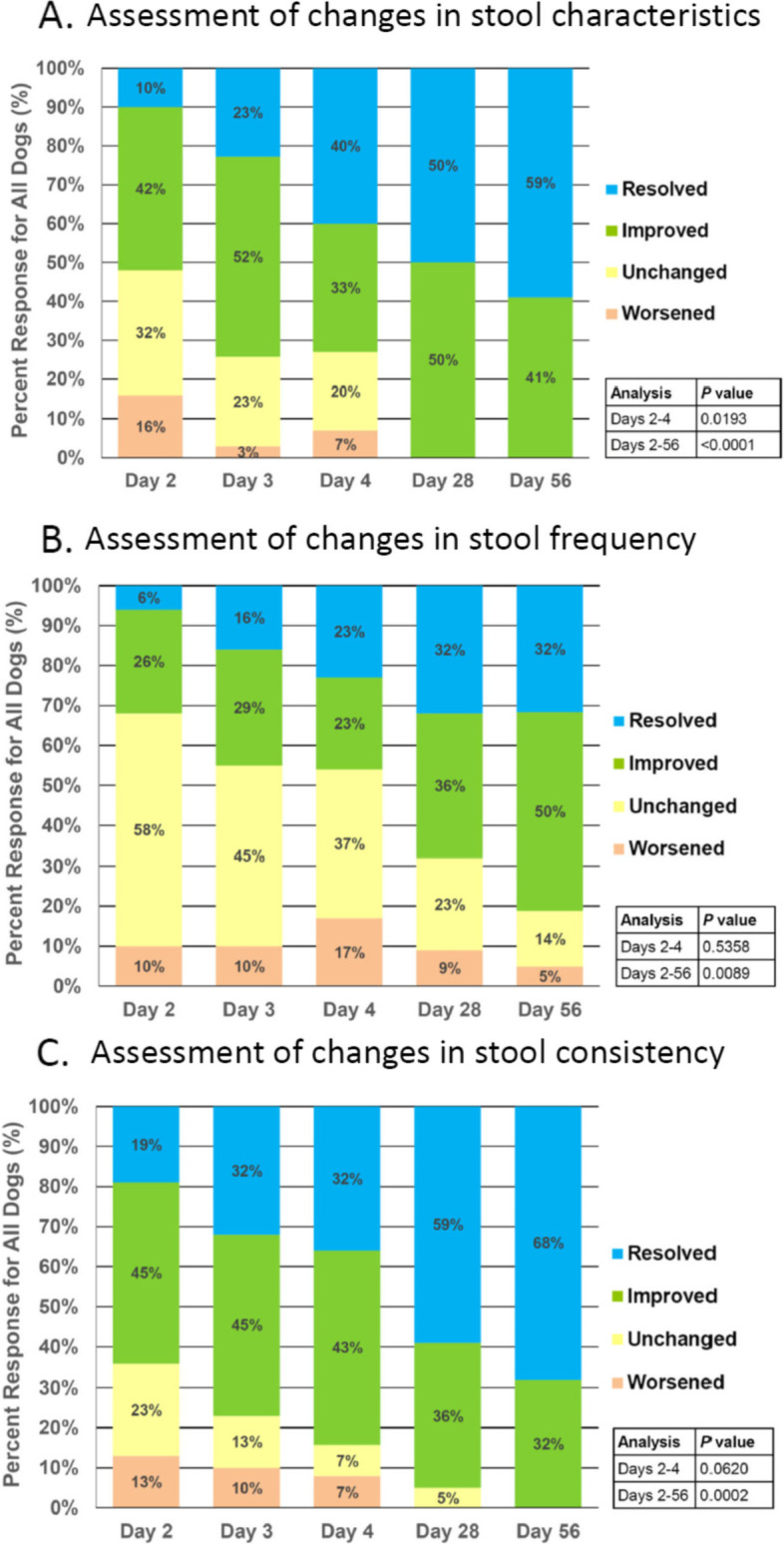
Veterinarian-reported response in (**A**) stool characteristics (presence of blood or mucus in stool), (**B**) stool frequency, and (**C**) stool consistency. *P* values represent comparisons of the distribution of responses at Day 4 or Day 56 versus Day 2

Changes in stool characteristics (e.g., presence of stool mucus and/or blood or presence of unusual odor, color, texture, and/or size) and stool frequency were evaluated by veterinarians while dogs were housed in the clinic (Days 2 to 4) and by owners when they returned home (Days 4 to 56) (Fig. 4). A significant improvement or a complete resolution of adverse stool characteristics or stool consistency was observed at Days 28 and 56. With respect to stool frequency, 50% of dogs showed improvement and 32% exhibited resolution of their increased stool frequency.

### Safety and tolerability

A total of 19 adverse events (AEs) were reported in 11 dogs during the trial, 16 of which were considered to be mild and most (12 of 19) were deemed to be unlikely to be related to the study intervention. The most commonly reported adverse event was vomiting, which was reported once in 5 dogs and twice in 1 dog. Two moderate AEs (chronic otitis externa/oily seborrhea and pain in the tail) were determined to be unrelated to the therapeutic food. On Day 6, one dog was euthanized for a previously undiagnosed heart condition, but this severe AE was also determined to be unrelated to the therapeutic food. Vital signs and blood parameters, including complete blood counts and blood chemistry (Supplemental Tables 1 and 2), were reviewed by a veterinarian and revealed no clinically significant changes throughout the duration of the study in all dogs.

### Medication use

Two dogs who had received metronidazole at enrollment were dismissed from the study, one on Day 3 and one on Day 5, based on low food intake. Two additional dogs received metronidazole starting on Day 3; however, both dogs completed the study through Day 56. A sensitivity analysis that excluded these dogs from the study demonstrated that the inclusion of these dogs did not impact the study’s outcomes or conclusions. Medications for conditions unrelated to chronic large bowel diarrhea were allowed and those prescribed during the study included the following: antianxiety medications (trazodone, 1 dog), pain medication (gabapentin and carprofen, 1 dog), dermatitis medications (florfenicol/terbinafine/betamethasone acetate, 1 dog; oclacitinib, 1 dog), and antibiotics (amoxicillin/clavulanate and neomycin/polymyxin B sulfate/dexamethasone drops, 1 dog; cefpodoxime, 2 dogs) for ocular or dermatological conditions.

## Discussion

Feeding the test food to dogs with chronic large bowel diarrhea significantly improved stool firmness and consistency within 24 h while progressively improving and resolving clinical signs over the course of the study. Based on veterinarians’ review of owner assessments, the majority of dogs (68%) with chronic large bowel diarrhea experienced complete resolution of their clinical signs, including poor stool consistency (68%), blood and mucus in stool (59%), and stool frequency (32%) when dogs were fed the study food for 56 days. Dog owners reported that their dogs had a significant decrease in the occurrence of nausea and vomiting and adverse stooling behaviors (straining, unproductive stooling attempts, and defecation accidents) in the 14 days after their dogs initiated the diet, the earliest time point evaluated by owners. Dog owners also reported a significant improvement in their pets’ QoL after their dogs had consumed the food for 28 days.

The therapeutic food evaluated in this study is a novel blend of whole grains, n-3 fatty acids, and prebiotic fibers, which contain bound polyphenols designed to nourish and activate the pet’s microbiome and promote digestive health. Several previous studies have established the benefits of foods containing similar fiber blends (pecan shells, flaxseed, pressed cranberries, beet pulp, and citrus pulp) when fed to companion pets [15, 21, 22]. In one study, dogs were fed the fiber blend in combination with two different base formulations, a hydrolyzed meat food and a grain rich food. Results from this study confirmed that the addition of the fiber blend into these foods provided several GI benefits, including improved stool consistency, lowered stool pH, increased beneficial gut microbes, and changes in microbial metabolites to indicate improved colonic health [15].

In another study in healthy adult cats, the fiber blend significantly increased fecal acetic and propionic acids, decreased putrefactive metabolites such as isobutyric, 2-methylbutyric, and isovaleric acids, increased moisture, and decreased pH compared with control food while maintaining acceptable stool scores [21]. Consistent with the canine study described above, the therapeutic food shifted the GI microbiome composition and metabolism of cats toward saccharolytic fermentation and decreased putrefactive metabolites, characteristics that may provide benefits for GI health [22].

In the current study, the therapeutic food was found to be safe and well tolerated for the duration of the study. The majority of AEs (16 of 19) were observed to be mild, while two moderate AEs were determined to be unrelated to the therapeutic food; one dog was euthanized due to a pre-existing, undiagnosed heart condition. Vital signs and blood parameters were found to be within normal clinical ranges for the duration of the study in all dogs.

The rapid improvement in signs observed following administration of the therapeutic food supports the use of nutritional intervention as a first-line therapy for dogs with chronic large bowel diarrhea. A food that incorporates a fiber blend with prebiotic content, water-holding and stool-bulking capacity, and plant fibers rich in polyphenols and antioxidants may be particularly useful in alleviating diarrhea and improving quality of life in this population. While antibiotics are frequently used as empirical treatment in dogs with chronic enteropathies [5], their use is not well supported by data from large, randomized, placebo-controlled trials [5, 23]. In addition, complications from acute and chronic administration of antibiotics remain a concern [5]. For example, broad-spectrum antibiotics, which can cause GI signs such as diarrhea in dogs, have been associated with disturbances in the microbiome and can be associated with resistant infections [5, 24–26]. In fact, antibiotics can induce dysbiosis by causing a rapid and significant decline in the richness, diversity, and evenness of the canine GI microbiome [27]. Therefore, the standard use of antibiotics to treat GI disease in dogs is not appropriate for all cases. Moreover, exposure to antibiotics has been implicated as a risk factor for the development of IBD [28, 29]. Because of the consequences of antibiotic therapy, nutrition interventions with specialized fiber sources are a preferred strategy for managing chronic diarrhea in dogs.

This study did have several limitations. First, the absence of a control group makes it difficult to ascertain whether the benefits we observed may have resulted from a time-related effect, rather than a dietary effect. However, given the persistent and pre-existing nature of the signs of chronic diarrhea in the dogs evaluated, we believe that it is unlikely that the improvements observed in this study were due solely to spontaneous resolution of their condition. Enrolled dogs were required to have had an active episode of diarrhea lasting at least 2 weeks or had been experiencing unresolved diarrhea or loose stools which, based on the veterinarian’s assessment, was likely to continue and become chronic in nature. Due to the duration and the severity of the dogs’ diarrhea, including a placebo control in the trial, would not have complied with standard animal welfare practices. The lack of a simple diagnostic test to confirm the precise cause of the dogs’ chronic large bowel diarrhea is another limitation. Veterinarians assessed the dogs based on their medical history, clinical signs, physical examination, and laboratory analyses, and did not conduct endoscopic biopsies on these patients and were thus unable to definitively diagnose the cause of the diarrhea in the pets in the study. Endoscopic biopsies were not conducted prior to enrollment for two reasons: (1) dietary interventions are typically recommended in dogs with chronic diarrhea before they undergo invasive diagnostic procedures; and (2) conducting invasive diagnostic procedures may subject patients to unnecessary discomfort and violates the Sponsor’s animal welfare standards for clinical studies. The sample size of the group was another potential limitation, as it may not be representative of the general canine population. However, given the fact that dogs enrolled in the trial were from geographically diverse locations that represented a range of regions in the continental US, we believe these results are generalizable to most dogs with non-infectious chronic diarrhea in the US.

## Conclusions

In this study, the therapeutic food rapidly improved stool consistency and resolved clinical signs in all dogs with chronic large bowel diarrhea, while dog owners reported improvement in stooling behaviors and QoL. A unique feature of this food was the fiber sources rich in antioxidant and anti-inflammatory compounds. Future work will examine how this novel fiber blend affects the microbiome of dogs over a longer period of time.

## Methods

### Study design

This was a prospective, single-arm, 8-week, multicenter clinical study of adult dogs with chronic large bowel diarrhea treated in private practices in the United States (US). The study was conducted between March 29, 2017, and March 20, 2018, at 11 general or specialty practices across all geographic regions of the continental US.

The study protocol was reviewed and approved by the Institutional Animal Care and Use Committee, Hill’s Pet Nutrition, Inc, in Topeka, Kansas (permit number, 719.0.0). Procedures were designed to avoid or minimize pain, discomfort, or distress, and dogs were monitored for any signs of disease. The dog’s health always took precedence over continuation in the trial in the case of an adverse event. Owners signed an informed consent form before their dog was enrolled and agreed to comply with the instructions given by the veterinarian.

### Patients

The eligibility of each dog was assessed by medical, drug, and dietary histories, physical examination, and laboratory analysis of blood and urine. Dogs were eligible for enrollment in the trial if they were aged 1 to 10 years, were treated in private practices in the US, and had been experiencing unresolved diarrhea (predominantly large bowel diarrhea) or loose stools which, based on the veterinarian’s assessment, was likely to continue. In addition to unresolved diarrhea or loose stools, the dogs must also have exhibited at least 1 of the following signs: frequent emission of feces; straining while defecating (dyschezia); frequent attempts to evacuate bowels (tenesmus); signs of abdominal discomfort; blood in stool (hematochezia); mucus in stool; vomiting; or loss of appetite. Dogs were also eligible if they had a current episode of diarrhea that had lasted at least 2 weeks or had a current episode of diarrhea with a history of persistent GI signs, including diarrhea or loose stools, for a minimum of 2 weeks.

On Study Day 1 (the enrollment visit), fecal float, fecal smear, and rectal cytology were performed to detect the presence of intestinal parasites and to help rule out other causes of diarrhea. Dogs were excluded from the trial if they had a known foreign body in their system, intestinal parasites, musculoskeletal issues, or any concurrent systemic disease. They were also excluded if they had a history of chronic use of medications that significantly influence colonic motility, if they had received oral antibiotics within 4 weeks, if they had been fed a therapeutic food within the past 3 months, or if they had a body mass index of less than 20 kg/m^2^ or more than 60 kg/m^2^. All dogs were assigned to the therapeutic food, Prescription Diet^®^ Gastrointestinal Biome™ Canine dry formula. The therapeutic food was packaged in unbranded 27-lb bags, which were distributed to investigators with feeding guidelines before the initiation of the study. Investigators and pet owners were blinded to the identity of the Sponsor and to the assigned therapeutic food. Dogs were switched to study food on Day 1 by clinic staff and veterinarians. Following discharge from the clinic, dog owners were instructed to continue feeding the study food exclusively on Day 4, to discontinue offering treats for the duration of the study, to avoid giving the study food to other pets, and to ensure that water was consistently available to the pet.

### Composition of therapeutic food

The therapeutic food (Hill's Prescription Diet Gastrointestinal Biome Dry Dog Food) is a nutritionally complete and balanced dry formulation that included whole grains (barley, corn, oats) and the following additional sources of prebiotics: ground pecan shells; cellulose; flaxseed; dried beet pulp; dried citrus pulp; pressed cranberries; dried pumpkin; and psyllium seed husks. The formulation also contained chicken, ginger root, fish oil, taurine, vitamins, and minerals. The nutritional composition of the food is described in more detail in Table 2. The prebiotic blend in the therapeutic food included 58.7 mg/g insoluble fiber and 6.4 mg/g of soluble fiber, for a total of 65.1 of dietary fiber and 205.8 mg/g of total polyphenols and provides a total Oxygen Radical Absorbance Capacity (ORAC) score of 3188.32.

**Table 2:** Composition of the therapeutic food with novel prebiotic fiber blend

Composition (%)	As Fed (per 100 kcal)	DMB
Protein	20	22
Fat	12	13
Crude Fiber	6	6
EPA	0.3	0.3
DHA	0.2	0.2
Sum n-3 Fatty Acids	1.3	1.4
Sum n-6 Fatty Acids	3.2	3.6
Prebiotic Fiber Blend	Beet Pulp, Citrus Pulp, Pressed Cranberries, Flaxseed, Pecan Shell Flour	

*DMB* Dry matter basis

### Study visits and procedures

Dogs that met criteria for enrollment were recruited upon presentation at the veterinary clinic. Following informed consent by owners, dogs were admitted to the clinic and housed on Days 1–3 of the study (Supplemental Table 3). Physical examinations, veterinary clinical evaluations, and collection of biological specimens, including blood and feces, were performed daily when dogs were housed at the veterinary clinic and during clinic visits. Eligible dogs were admitted to the clinic, began consuming the study food, and remained in the clinic for veterinary observation and care until they were discharged on Study Day 4. Three owners chose to opt out of the in-clinic stay and took their dogs home. Pet owners who opted out of the clinic stay agreed to return to the clinic with their dogs on Days 1 to 4 and stayed in the clinic each day until fecal and blood samples were obtained. Pet owners who did not leave their dogs in the veterinary clinic overnight completed the stool diary on Days 2 through 4. During the first 3 days after enrollment (Study Days 1–4), the clinic staff recorded food intake and completed a stool diary, including stool frequency, consistency, and characteristics (Appendices A, B, and C). Stools were collected, processed, and frozen for later analysis of the following: moisture, microbiota composition, SCFAs, pH, ash, minerals, and metabolomics. Dog owners evaluated stool consistency prior to enrollment and recorded stooling behaviors at baseline. Dog owners also completed an Initial Gastrointestinal Behavior Questionnaire, Pet Owner Food Questionnaire, and a Quality of Life (QoL) Questionnaire [30]**.** After discharge on Day 4, dog owners completed a daily stool log to document stool consistency and stool characteristics, recorded stooling behaviors, and submitted a food and QoL questionnaire on study Days 14, 28 and 56.

Dogs were removed from the study if diarrhea did not improve within 7 days and the veterinarian determined that the patient’s health would improve if it were released from the study or if a diagnosis warranted an increase of approved pre-study medication. To ensure the welfare of the patients, dogs were also removed from the study if food intake was less than 60% of their maintenance energy requirements during Study Days 1–5, if they did not eat for more than 36 h during Study Days 1–3, or if they had not defecated at least once per day during their stay at the clinic. Dog owners were compensated for their dog’s participation.

### Study endpoints

The primary outcome of the study was owner and veterinary assessment of stool consistency. Veterinarians based their assessments on information provided by dog owners regarding stool consistency, frequency, and characteristics (presence of stool mucus and/or blood or presence of unusual odor, color, texture, and/or size). Stool consistency was graded on a 5-point scale, from grade 1 (liquid stools) to grade 5 (firm, formed stools). Dog owners evaluated nausea-related behavior, vomiting, lethargy, and stooling behaviors (straining, unproductive stooling attempts, and defecation accidents) on a 0 (never) to 100 (always) visual analog scale. Dogs’ QoL was also assessed on a 0 (very poor) to 10 (excellent) visual analog scale. When veterinarians rated the overall clinical response for each dog, they considered the physical exam, their veterinary clinical evaluation (VCE), and also included clinical signs exhibited by the dog (observed by the dog owner and veterinarian), along with any changes in medications, treatments or procedures provided to the patient. The clinical response of dogs was classified based on the following definitions: 1) a negative response was defined as an increase in the frequency and/or severity of signs or symptoms of chronic large bowel diarrhea and inflammatory enteropathies from the start of the study (worsened); 2) a non-response was defined as no change in the frequency and/or severity of signs from the start of the study (unchanged); 3) a positive response was defined as a decrease in the frequency and/or severity of signs from the start of the study (improved); and 4) a complete response was defined as a complete resolution of the signs of large bowel diarrhea and inflammatory enteropathy (resolved). Relapse was defined as the return of signs or symptoms after the signs or symptoms had completely resolved.

Secondary outcomes included stooling behaviors, complete blood counts and blood chemistry, and QoL. Veterinarians also monitored dogs for the recurrence of the signs and symptoms of large bowel diarrhea and inflammatory enteropathy. Adverse events and medication use were also documented.

### Statistical data analysis

The number of patients targeted for the study was 30 dogs, which was the estimated number needed to detect a statistically significant difference in the clinical outcomes. This estimate was based on the results of previous analyses that collected information about the hypothesis under investigation and assumed a nominal power of 0.9, a *P* value of 0.05, and a dismissal rate of 20% [15].

Scores from the veterinary clinical evaluations were analyzed using a general linear model with study day as the fixed effect. The correlation between the repeated measures was modeled using the REPEATED option in SAS^®^ PROC MIXED. To account for the correlation between the repeated measurements on each patient over time, the appropriate variance–covariance structure for each response variable was selected using the Akaike Information Criterion and Bayesian Information Criterion fit statistics. With the assessment of overall clinical signs associated with chronic diarrhea, mean scores from all post Day 1 time points were compared against Day 1 mean scores using a lower one-sided t-test to determine whether there was a significant reduction in overall clinical signs compared to Day 1. With the assessment of overall clinical signs associated with chronic diarrhea since the previous exam, and overall clinical signs associated with chronic diarrhea since the start of the study, a score of 50 (which represented no change on the 1–100 scale) was subtracted from each score, and a two-sided t-test was used to test whether the mean change was significantly different from 0.

Stool consistency scores were also analyzed using a first-order autoregressive heterogeneous covariance structure to account for correlation between the repeated measurements on each patient. Prior to the Day 1 enrollment, owners reported a stool score for their dogs. All mean scores after Day 1 were compared against the mean scores at Day 1 using a two-sided t-test.

Ordered categorical scores (worsened, unchanged, improved, resolved) for veterinarian assessments of clinical response, physical exam, stool characteristics, stool consistency, and stool frequency were analyzed using the Cochran-Mantel–Haenszel test using modified ridit scores with Day as the row variable and categorical score as the column variable. Modified ridit scores produce van Elteren’s extension of the Wilcoxon rank sum test [30].

Scores from dog owner assessments of nausea/vomiting, straining, unproductive attempts to defecate, lethargy, defecation accidents, and QoL were analyzed using the same linear model as described above. Means scores at Days 14, 28, and 56 were compared against scores at Day 1 using a two-sided t-test. All analyses were performed using SAS^®^, Version 9.4. Statistical differences were deemed statistically significant if *P* values were < 0.05.

## Supplementary Information

**Additional file  1:** **Table  1. **Complete blood count (CBC) results on Days 1, 28, and 56 with laboratory reference ranges. Data are presented as means ± standard deviations. 

**Additional file 2:** **Table 2.** Serum chemistry results on Days 1, 28, and 56 with laboratory reference ranges. Data are presented as means ± standard deviations.

**Additional file  3:** **Table  3.** Study visits and procedures.

**Additional file 4:**  **Appendix A.** Initial Gastrointestinal Behavior Questionnaire.

**Additional file 5:** **Appendix B. **Stool Consistency Grading Scale.

**Additional file 6:** **Appendix C. **Veterinary Clinical Evaluation.

## Data Availability

All data generated and/or analyzed during this study are included in this published article and its supplementary information files.

## Abbreviations

AE: Adverse event
DHA: Docosahexaenoic acid
EPA: Eicosapentaenoic acid
GI: Gastrointestinal
IBD: Inflammatory bowel disease
QoL: Quality of life
SCFA: Short chain fatty acid
SE: Standard error
US: United States
