# Supplementary material for: A prospective multicenter study of the efficacy of a fiber-supplemented dietary intervention in dogs with chronic large bowel diarrhea
Source: BMC Vet Res. 2022 Jun 24;18:244. doi: 10.1186/s12917-022-03302-8 (PMC9229818; doi:10.1186/s12917-022-03302-8)
Supplement: Supplementary file 1 — Additional file 1: Table 1. Complete blood count (CBC) results on Days 1, 28, and 56 with laboratory reference ranges. Data are presented as means ± standard deviations. [file 12917_2022_3302_MOESM1_ESM.docx]

**SUPPLEMENTAL MATERIALS**

**Table 1.** Complete blood count (CBC) results on Days 1, 28, and 56 with laboratory reference ranges. Data are presented as means ± standard deviations.

|  | **Reference Range** | **Day 1** | **Day 28** | **Day 56** |
| --- | --- | --- | --- | --- |
| Absolute basophils (1000/µL) | 0.00 – 0.10 | 0.028 ± 0.075 | 0.000 ± 0.000 | 0.000 ± 0.000 |
| Absolute eosinophils (1000/µL) | 0.00 – 1.40 | 0.566 ± 0.460 | 0.546 ± 0.447 | 0.436 ± 0.285 |
| Absolute lymphocytes (1000/µL) | 0.30 – 3.90 | 1.793 ± 0.759 | 2.050 ± 0.768 | 2.158 ± 0.854 |
| Absolute monocytes (1000/µL) | 0.00 – 1.40 | 0.478 ± 0.225 | 0.477 ± 0.198 | 0.418 ± 0.178 |
| Absolute segments (1000/µL) | 2.50 – 15.70 | 7.06 ± 2.85 | 7.14 ± 3.20 | 7.27 ± 2.59 |
| White blood cells (1000/µL) | 4.0 – 18.2 | 9.93 ± 2.89 | 10.21 ± 3.68 | 10.32 ± 2.98 |
| Red blood cells (M/ µL) | 4.48 – 8.53 | 7.133 ± 0.908 | 7.340 ± 0.745 | 7.267 ± 0.799 |
| Platelets (1000/µL) | 140 - 540 | 362.8 ± 147.8 | 323.3 ± 152.3 | 382.5 ± 160.2 |
| Mean corpuscular hemoglobin (pg) | 21.0 – 27.0 | 24.48 ± 1.28 | 24.54 ± 1.25 | 24.76 ± 1.35 |
| Mean corpuscular hemoglobin concentration (g/dL) | 30.1 – 41.9 | 33.56 ± 2.17 | 34.26 ± 1.96 | 34.66 ± 2.10 |
| Mean corpuscular volume (fL) | 63.0 – 78.3 | 73.10 ± 4.40 | 71.71 ± 3.30 | 71.55 ± 3.92 |
| Hemoglobin (g/dL) | 10.5 – 20.1 | 17.47 ± 2.46 | 18.02 ± 2.15 | 17.99 ± 2.11 |
| Hematocrit (%) | 33.0 – 58.7 | 51.96 ± 5.71 | 52.60 ± 5.53 | 51.92 ± 5.69 |
| Red cell distribution width (fL) | 12.9 – 21.0 | 15.62 ± 1.85 | 16.21 ± 1.67 | 16.23 ± 1.83 |
