# Supplementary material for: A prospective multicenter study of the efficacy of a fiber-supplemented dietary intervention in dogs with chronic large bowel diarrhea
Source: BMC Vet Res. 2022 Jun 24;18:244. doi: 10.1186/s12917-022-03302-8 (PMC9229818; doi:10.1186/s12917-022-03302-8)
Supplement: Supplementary file 2 — Additional file 2: Table 2. Serum chemistry results on Days 1, 28, and 56 with laboratory reference ranges. Data are presented as means ± standard deviations. [file 12917_2022_3302_MOESM2_ESM.docx]

**SUPPLEMENTAL MATERIALS**

**Table 2.** Serum chemistry results on Days 1, 28, and 56 with laboratory reference ranges. Data are presented as means ± standard deviations.

|  | **Reference Range** | **Day 1** | **Day 28** | **Day 56** |
| --- | --- | --- | --- | --- |
| Albumin (g/dL) | 2.6 – 4.0 | 3.53 ± 0.35 | 3.51 ± 0.60 | 3.57 ± 0.28 |
| Globulin (g/dL) | 2.0 – 3.8 | 2.78 ± 0.55 | 2.80 ± 0.54 | 2.80 ± 0.45 |
| Albumin/globulin ratio |  | 1.30 ± 0.25 | 1.29 ± 0.28 | 1.30 ± 0.23 |
| Alkaline phosphatase (U/L) | 13 – 289 | 38.2 ± 24.7 | 58.9 ± 48.1 | 68.7 ± 66.2 |
| Alanine aminotransferase (U/L) | 14 – 151 | 39.5 ± 20.1 | 40.4 ± 14.9 | 41.7 ± 13.6 |
| Aspartate aminotransferase (U/L) | 18 – 86 | 28.5 ± 8.7 | 27.2 ± 5.1 | 27.1 ± 5.4 |
| Gamma-glutamyl transferase (U/L) | 3 – 19 | 4.0 ± 1.7 | 4.3 ± 2.9 | 4.0 ± 1.9 |
| Blood urea nitrogen (mg/dL) | 8 – 30 | 16.14 ± 3.34 | 14.10 ± 4.16 | 14.60 ± 2.41 |
| Creatinine (mg/dL) | 0.4 – 2.0 | 0.905 ± 0.311 | 0.790 ± 0.159 | 0.750 ± 0.157 |
| Blood urea nitrogen/creatinine ratio |  | 19.71 ± 7.68 | 19.05 ± 5.75 | 20.65 ± 7.24 |
| Total bilirubin | 0.0 – 0.5 | 0.114 ± 0.091 | 0.110 ± 0.064 | 0.125 ± 0.091 |
| Calcium (mg/dL) | 8.7 – 12.0 | 9.88 ± 0.52 | 9.73 ± 1.27 | 10.04 ± 0.46 |
| Magnesium (mg/dL) | 1.6 – 2.3 | 2.05 ± 0.22 | 2.14 ± 0.16 | 2.07 ± 0.17 |
| Potassium (mmol/L) | 3.4 – 5.6 | 4.53 ± 0.39 | 4.76 ± 0.44 | 4.57 ± 0.38 |
| Sodium (mmol/L) | 141 – 159 | 148 ± 3.27 | 147 ± 2.44 | 147 ± 2.63 |
| Sodium/potassium ratio |  | 33.0 ± 3.1 | 31.2 ± 3.0 | 32.4 ± 3.0 |
| Chloride (mmol/L) | 100 – 121 | 113.33 ± 3.53 | 112.71 ± 3.27 | 112.95 ± 2.50 |
| Anion gap (mmol/L) | 17 – 28 | 19.6 ± 3.3 | 19.6 ± 2.7 | 18.3 ± 2.3 |
| CO_2_ (mmol/L) | 16 – 31 | 19.6 ± 2.4 | 20.0 ± 2.0 | 20.4 ± 2.9 |
| Phosphorus (mg/dL) | 2.5 – 7.9 | 4.27 ± 0.77 | 3.82 ± 0.80 | 4.08 ± 0.53 |
| Glucose (mg/dL) | 74 – 145 | 94.0 ± 18.2 | 90.3 ± 19.5 | 97.7 ± 11.6 |
| Total protein (g/dL) | 5.0 – 8.3 | 6.31 ± 0.73 | 6.31 ± 0.88 | 6.37 ± 0.49 |
| Cholesterol (mg/dL) | 98 – 300 | 191.1 ± 51.4 | 201.7 ± 51.4 | 218.5 ± 62.9 |
| Triglycerides (mg/dL) | 36 – 240 | 102.0 ± 83.6 | 132.4 ± 114.2 | 141.3 ± 212.9 |
