# Supplementary figures and images for: A prospective multicenter study of the efficacy of a fiber-supplemented dietary intervention in dogs with chronic large bowel diarrhea
Source: BMC Vet Res. 2022 Jun 24;18:244. doi: 10.1186/s12917-022-03302-8 (PMC9229818; doi:10.1186/s12917-022-03302-8)

**SUPPLEMENTAL MATERIALS**

**Table 3.** Study visits and procedures


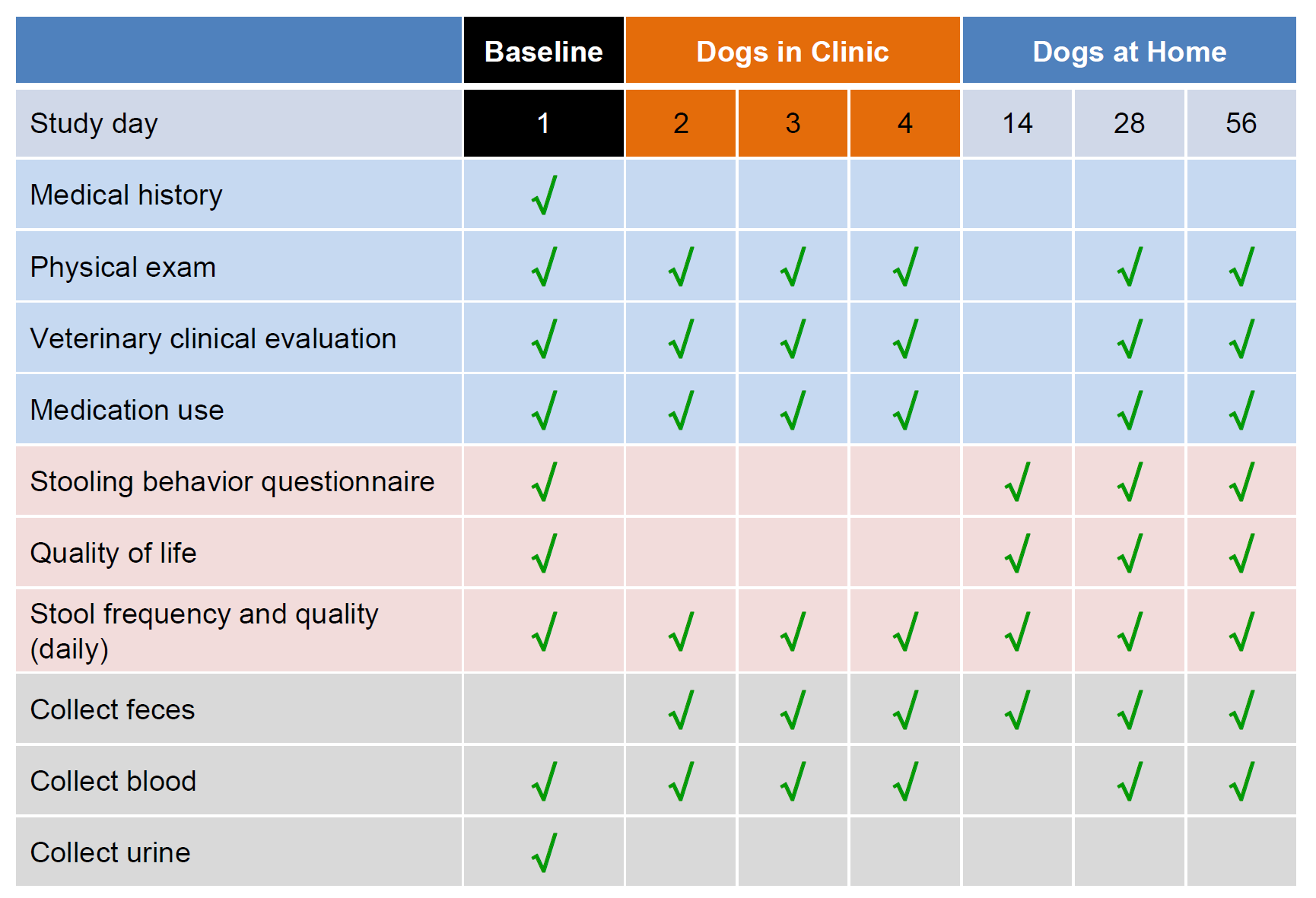

Supplement: Supplementary file 3 — Additional file 3: Table 3. Study visits and procedures. [file 12917_2022_3302_MOESM3_ESM.docx]
