# Supplementary material for: A prospective multicenter study of the efficacy of a fiber-supplemented dietary intervention in dogs with chronic large bowel diarrhea
Source: BMC Vet Res. 2022 Jun 24;18:244. doi: 10.1186/s12917-022-03302-8 (PMC9229818; doi:10.1186/s12917-022-03302-8)

**APPENDICES**

**Appendix A: Initial Gastrointestinal Behavior Questionnaire**

**Initial Gastrointestinal Behavior Questionnaire**

**Thinking about the current condition of your pet’s stooling behavior, please answer the following by marking the box which best represents your assessment of the following statements.**

***Defecation is defined as the discharge of stools from the body.**

Check the box below at the point that best describes **HOW OFTEN YOU ARE ABLE TO OBSERVE YOUR PET ON MOST DAYS.**

[ ] Check this box if unable to assess

[ ] More than two hours

[ ] 1-2 hours

[ ] 30-60 minutes

[ ] 15-30 minutes

[ ] Less than 15 minutes

Check the box below at the point that best describes **HOW OFTEN YOUR PET CURRENTLY DEFECATES.**

[ ] Check this box if unable to assess

[ ] Less than once a week

[ ] Twice a week

[ ] Every other day

[ ] Once a day

[ ] Twice a day

[ ] 3-5 times a day

[ ] 6-9 times a day

[ ] 10 or more times a day

Check the box below at the point that best describes **HOW OFTEN YOUR PET ACTS NAUSEOUS AND/OR VOMITS.**

[ ] Check this box if unable to assess.
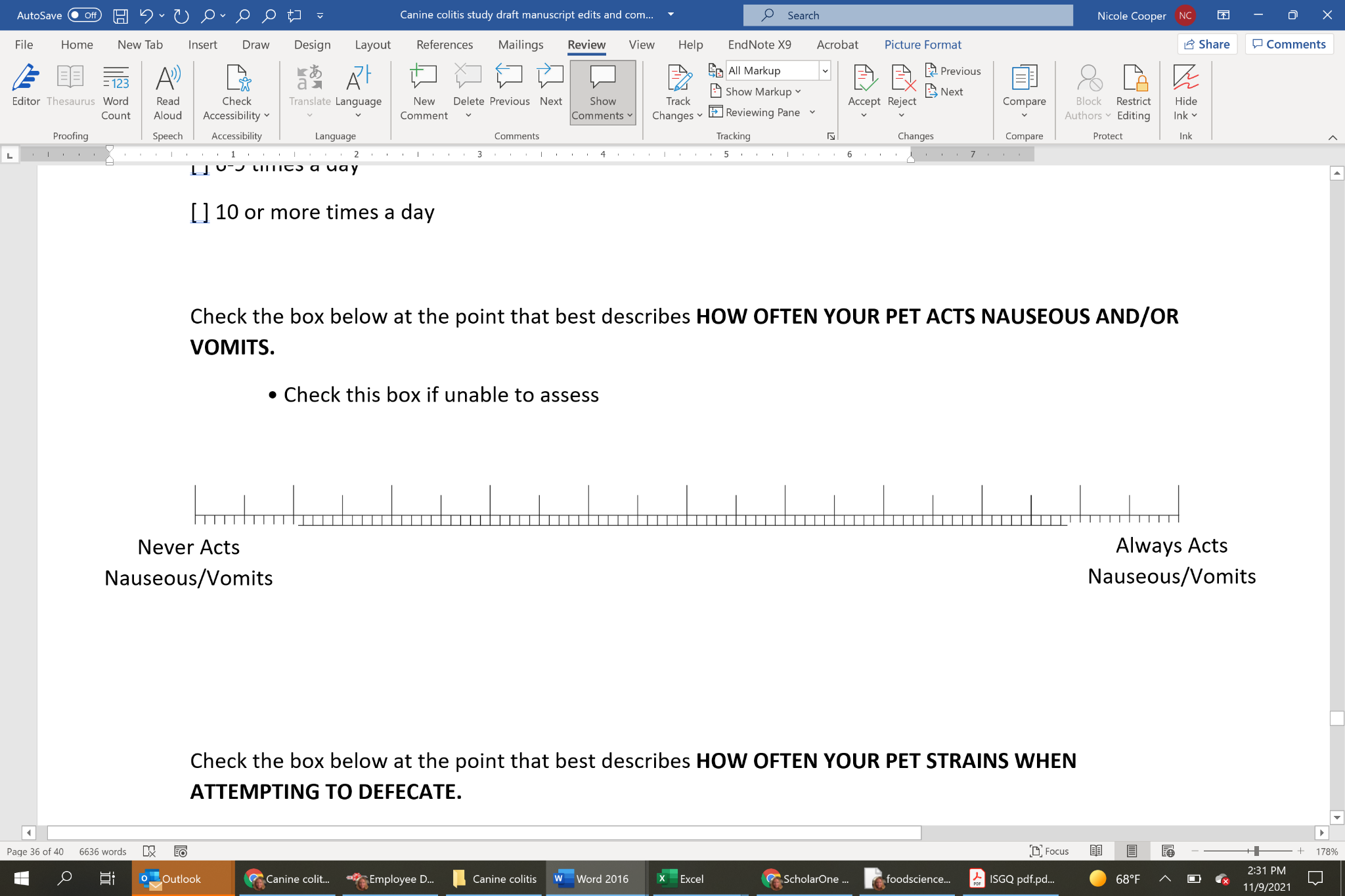
ess

Check the box below at the point that best describes **HOW OFTEN YOUR PET STRAINS WHEN ATTEMPTING TO DEFECATE.**

[ ] Check this box if unable to assess


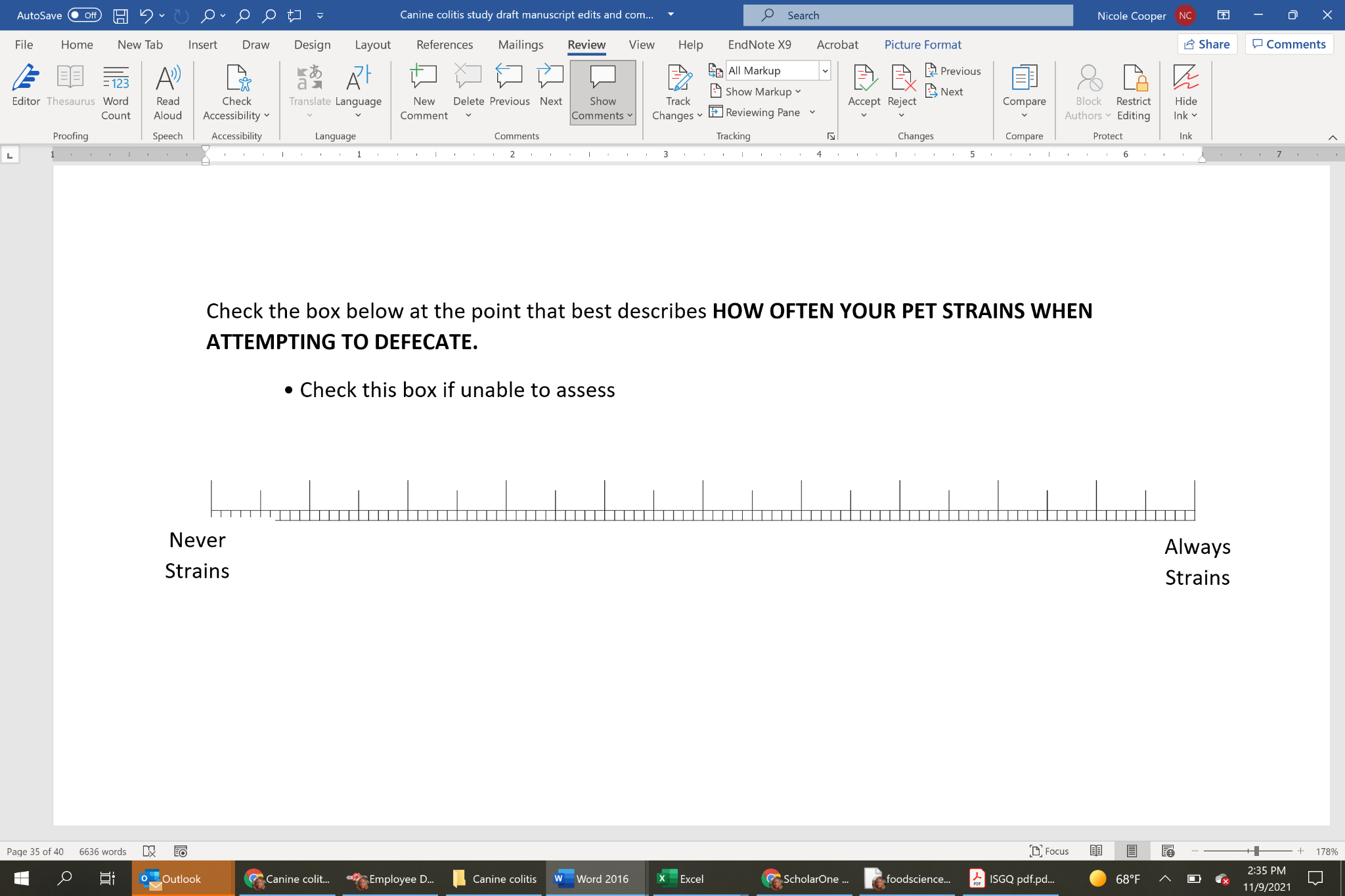


Check the box below at the point that best describes **HOW OFTEN YOUR PET MAKES UNPRODUCTIVE ATTEMPTS TO DEFECATE.**

[ ] Check this box if unable to assess


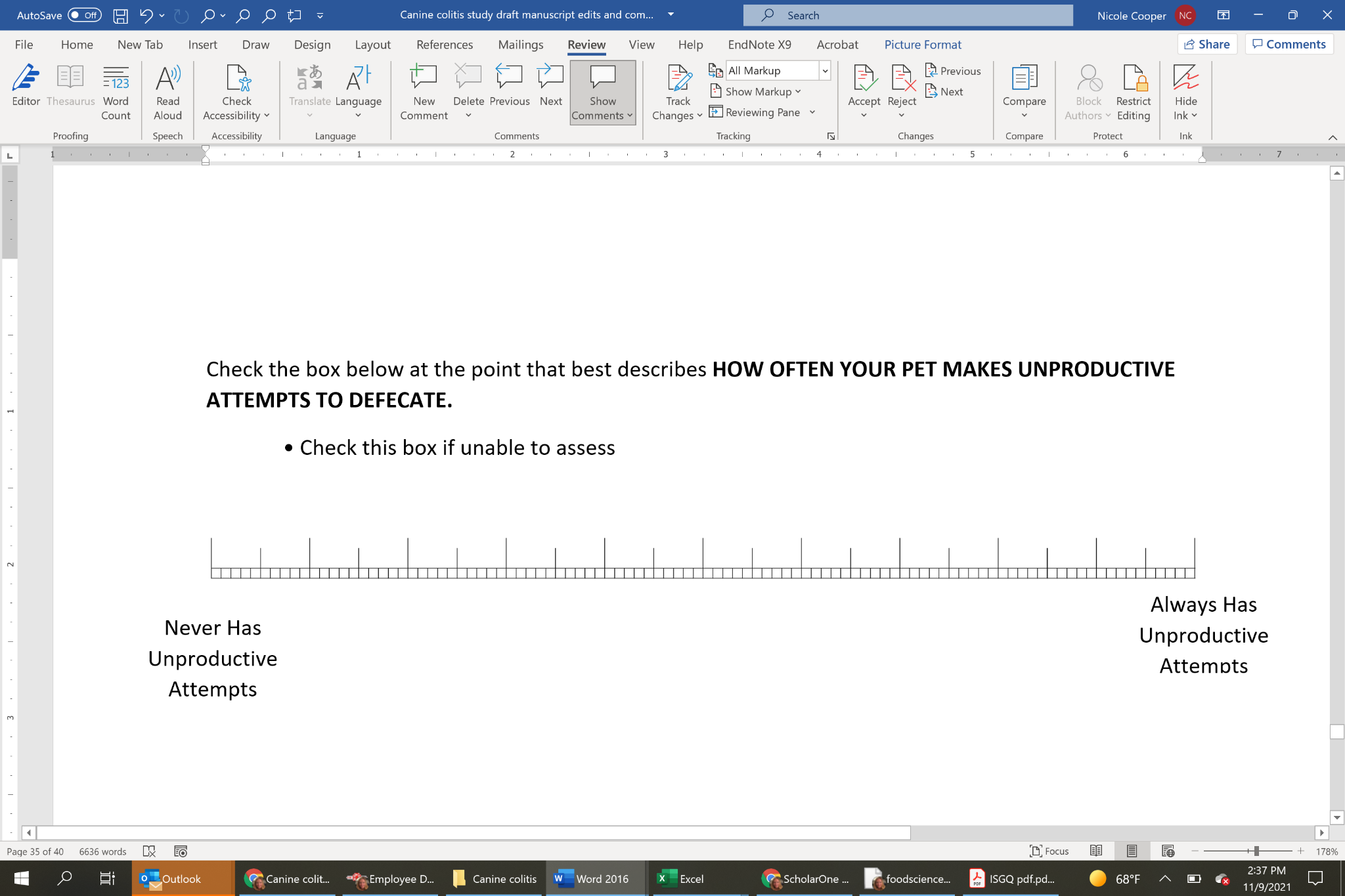


Check the box below at the point that best describes **HOW OFTEN YOUR PET IS LETHARGIC.**

[ ] Check this box if unable to assess


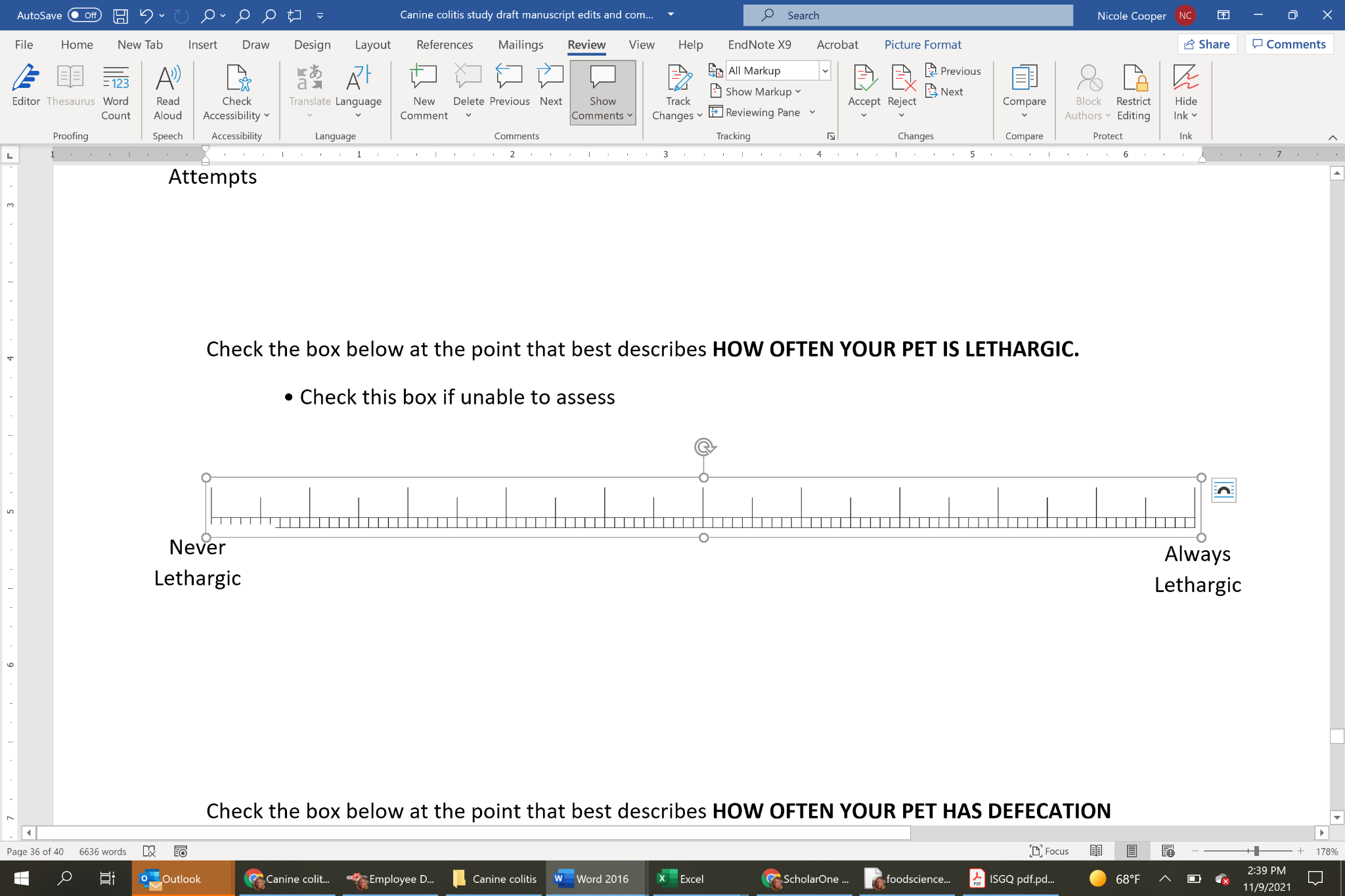


Check the box below at the point that best describes **HOW OFTEN YOUR PET HAS DEFECATION ACCIDENTS.**

[ ] Check this box if unable to assess


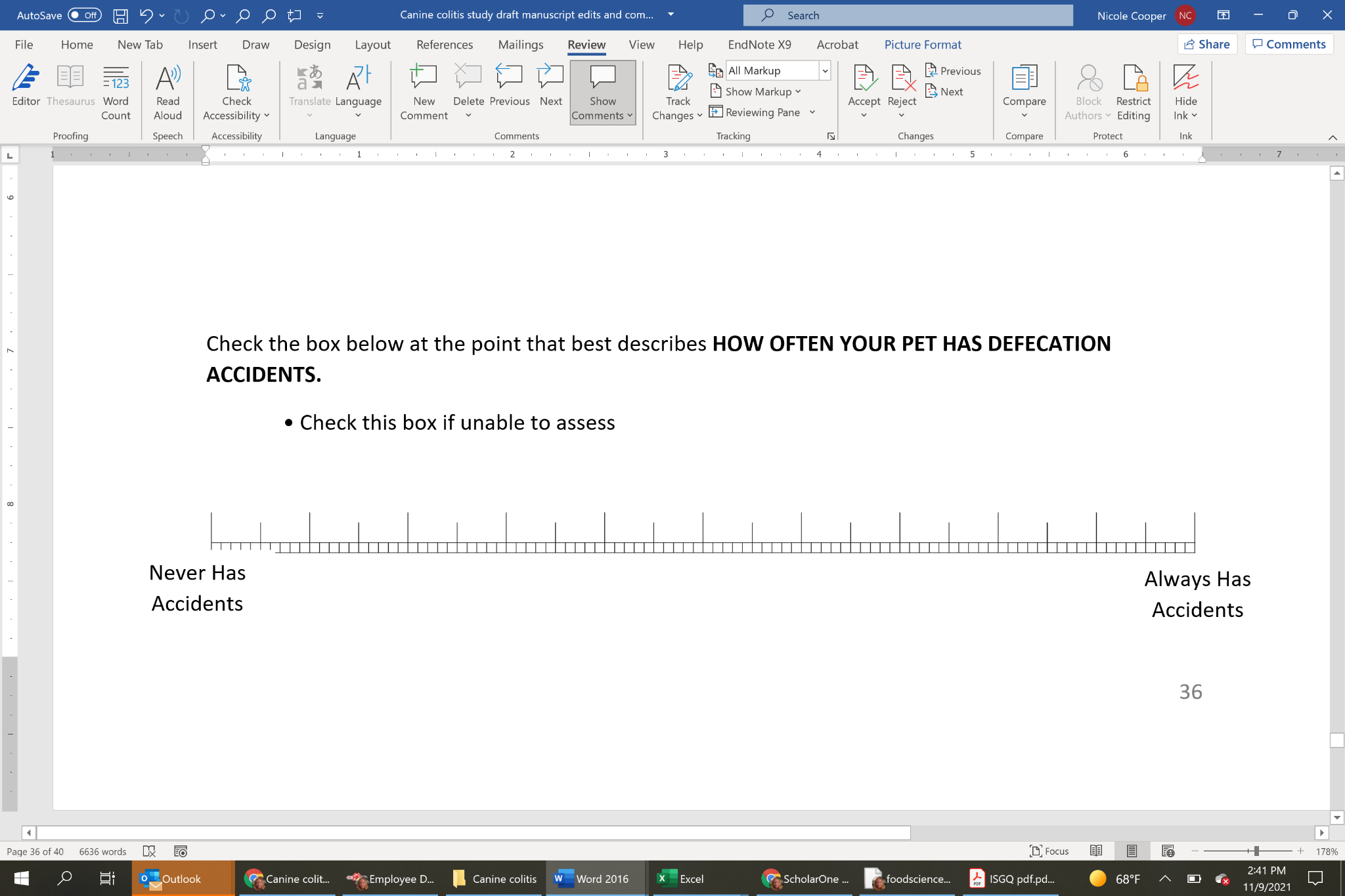

Supplement: Supplementary file 4 — Additional file 4: Appendix A. Initial Gastrointestinal Behavior Questionnaire. [file 12917_2022_3302_MOESM4_ESM.docx]
