# Supplementary material for: A prospective multicenter study of the efficacy of a fiber-supplemented dietary intervention in dogs with chronic large bowel diarrhea
Source: BMC Vet Res. 2022 Jun 24;18:244. doi: 10.1186/s12917-022-03302-8 (PMC9229818; doi:10.1186/s12917-022-03302-8)

**Appendix B: Stool Consistency Grading Scale**

Veterinarians used the following Stool Consistency Grading Scale to evaluate the stool consistency of dogs enrolled in the study.


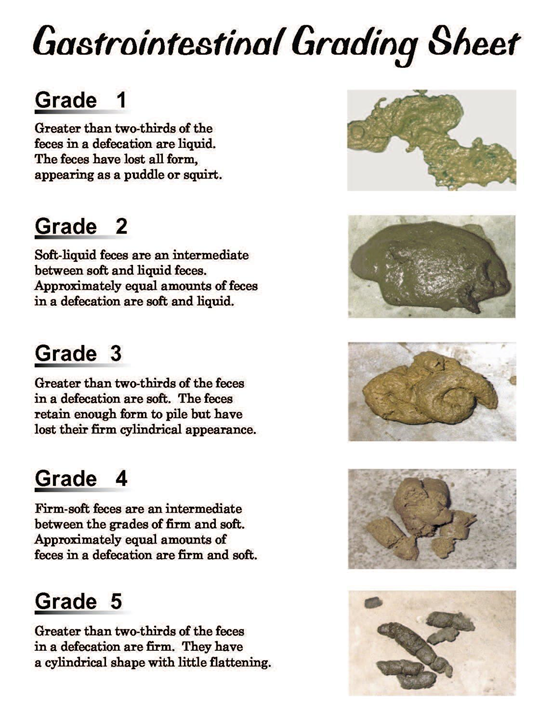

Supplement: Supplementary file 5 — Additional file 5: Appendix B. Stool Consistency Grading Scale. [file 12917_2022_3302_MOESM5_ESM.docx]
