# Supplementary material for: A prospective multicenter study of the efficacy of a fiber-supplemented dietary intervention in dogs with chronic large bowel diarrhea
Source: BMC Vet Res. 2022 Jun 24;18:244. doi: 10.1186/s12917-022-03302-8 (PMC9229818; doi:10.1186/s12917-022-03302-8)
Supplement: Supplementary file 6 — Additional file 6: Appendix C. Veterinary Clinical Evaluation. [file 12917_2022_3302_MOESM6_ESM.docx]

**Appendix C: Veterinary Clinical Evaluation**

**Veterinary Clinical Evaluation**

Please indicate which of the following were administered **during today’s visit** (check all that apply). Update the Concomitant Medications form as needed.

- Check here if none of the following were administered
- Antacid
- Antibiotic
- Antidiarrheal agent
- Anticholinergic medication
- Antimuscarinic agent
- Antispasmodic agent (ex, aminopentamide)
- Biopsy - Large intestinal
- Biopsy - Small intestinal
- Corticosteroid
- IV fluids
- Non-steroidal anti-inflammatory (NSAID)
- Opioid pain medication
- Other pain medication
- Probiotics
- X-ray
- Other (specify)__________________________________________________
- Other (specify)__________________________________________________
- Other (specify)__________________________________________________

Draw a single vertical line at the point that best describes **THE OVERALL CLINICAL SIGNS ASSOCIATED WITH COLITIS TODAY.**

- Check this box if unable to assess


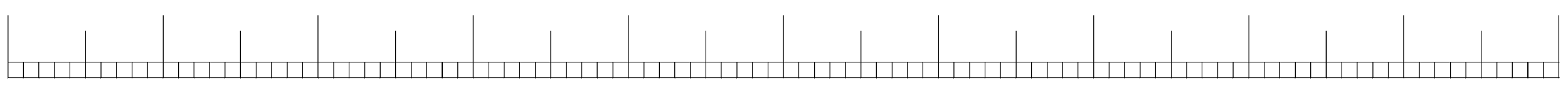


Severe Clinical Signs

No Clinical Signs

Moderate Clinical Signs

Draw a single vertical line at the point that best describes **HOW THE OVERALL CLINICAL SIGNS ASSOCIATED WITH COLITIS HAVE CHANGED SINCE THE PREVIOUS EXAM.**

- Check this box if unable to assess


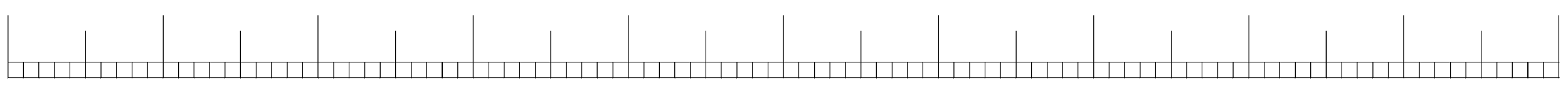


Significant Deterioration

Deterioration

Significant Improvement

No Change

Draw a single vertical line at the point that best describes **HOW THE OVERALL CLINICAL SIGNS ASSOCIATED WITH COLITIS HAVE CHANGED SINCE THE START OF THE STUDY.**

- Check this box if unable to assess


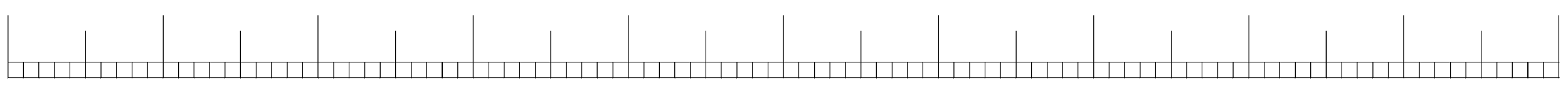


Significant Deterioration

Deterioration

Significant Improvement

No Change

**Based on the clinical signs associated with colitis in this dog today, I recommend the following changes to the dog’s medications/supplements:**

- Check this box if **no changes are recommended to any medications/supplements. Skip to next question**
- Check this box if **changes are recommended to at least one medications/supplement (including starting/discontinuing)** and specify which medications/supplements are being changed below. You must complete the section below and update the Concomitant Medications form if this box is checked.

**Name of Medication/Supplement**: _________________________________________________________________

Check one:

- New medication (update Concomitant Medications form)
- Increase (update Concomitant Medications form)
- Decrease (update Concomitant Medications form)
- Discontinue medication (update Concomitant Medications form)
- Other (specify and update Concomitant Medications form as needed) _________________________

_________________________________________________________________________________

**Name of Medication/Supplement**: _________________________________________________________________

Check one:

- New medication (update Concomitant Medications form)
- Increase (update Concomitant Medications form)
- Decrease (update Concomitant Medications form)
- Discontinue medication (update Concomitant Medications form)
- Other (specify and update Concomitant Medications form as needed) _________________________

_________________________________________________________________________________

**Name of Medication/Supplement:** _________________________________________________________________

Check one:

- New medication (update Concomitant Medications form)
- Increase (update Concomitant Medications form)
- Decrease (update Concomitant Medications form)
- Discontinue medication (update Concomitant Medications form)
- Other (specify and update Concomitant Medications form as needed) _________________________

_________________________________________________________________________________

**Name of Medication/Supplement:** _________________________________________________________________

Check one:

- New medication (update Concomitant Medications form)
- Increase (update Concomitant Medications form)
- Decrease (update Concomitant Medications form)
- Discontinue medication (update Concomitant Medications form)
- Other (specify and update Concomitant Medications form as needed) _________________________

_________________________________________________________________________________

**Based on the clinical signs associated with colitis in this dog today, I recommend the following changes to the dog’s treatments/procedures:**

- Check this box if **no changes are recommended to any treatments/procedures. Skip to next question**
- Check this box if **changes are recommended to at least one treatment/procedure (including starting/discontinuing)** and specify which treatments/procedures are being changed below.

**Name of Treatment/Procedure**: _________________________________________________________________

Check one:

- New treatment/procedure
- Increase
- Decrease
- Discontinue treatment/procedure
- Other (specify) ________________________________________________________________________

**Name of Treatment/Procedure**: _________________________________________________________________

Check one:

- New treatment/procedure
- Increase
- Decrease
- Discontinue treatment/procedure
- Other (specify) ________________________________________________________________________

**Name of Treatment/Procedure**: _________________________________________________________________

Check one:

- New treatment/procedure
- Increase
- Decrease
- Discontinue treatment/procedure
- Other (specify) ________________________________________________________________________

**Name of Treatment/Procedure**: _________________________________________________________________

Check one:

- New treatment/procedure
- Increase
- Decrease
- Discontinue treatment/procedure
- Other (specify) ________________________________________________________________________

Based on the information above and the dog’s overall condition, do you feel that the dog’s clinical response today **compared to the start of the study** can be classified as negative, non-response, positive, complete response, or recurrence?

- **Negative Response** (a negative response is defined as an **increase compared to the start of the study** in frequency and/or severity of signs).
- **Non-Response** (a non-response is defined as **no change compared to the start of the study** in frequency and/or severity of signs).
- **Positive Response** (a positive response is defined as a **decrease compared to the start of the study** in the frequency and/or severity of signs).
- **Complete Response** (is defined as a **complete resolution compared to the start of the study** in frequency and/or severity of signs).
- **Recurrence (**is defined as a **complete resolution compared to the start of the study** in frequency and/or severity of signs **followed by an increase in frequency and/or severity of signs**)

Please explain your answer by checking the appropriate boxes:

Responses are required for all fields except Other.

|  | **Today compared to study start** | | | |
| --- | --- | --- | --- | --- |
| Parameter | Worsened | Unchanged | Improved | Resolved |
| Stool consistency |  |  |  |  |
| Stool frequency |  |  |  |  |
| Stool characteristics |  |  |  |  |
| Physical exam |  |  |  |  |
| Other (specify)____________________ |  |  |  |  |
| Other (specify)_____________________ |  |  |  |  |
| Other (specify)____________________  Allowed to add additional Other fields as needed |  |  |  |  |

Responses are required only if Concomitant Medications form has been completed, field should populate but responses are not required if Concomitant Medications form has not been completed.

|  |  | **Compared to study start** | | | |  |
| --- | --- | --- | --- | --- | --- | --- |
| Parameter | New | Increase | Unchanged | Decrease | Discontinue | Other (specify) |
| Use of Medication/Supplement (specify and complete Concomitant Medications form)_________________________  _____________________________________________  Allowed to add additional Medication/Supplement fields as needed |  |  |  |  |  | _______________________ |
| Use of Treatment/Procedure (specify)_________________________  ___________________________________________  ___________________________________________  Allowed to add additional Treatment/Procedure fields as needed |  |  |  |  |  | _______________________ |
